# Surgical Management of an Intracranial Injury Caused by a Handcrafted Arrow in Sub-Saharan Africa: A Global Neurosurgical Relevance

**DOI:** 10.7759/cureus.86374

**Published:** 2025-06-19

**Authors:** Allie L Heineman, Quinn Jackson, Chris Karas, Robert Galler, Victor Awuor, Hiren Patel

**Affiliations:** 1 Medicine, Nova Southeastern University Dr. Kiran C. Patel College of Osteopathic Medicine, Fort Lauderdale, USA; 2 Neurosurgery, Cleveland Clinic Hillcrest Hospital, Mayfield Heights, USA; 3 Neurosurgery, Northwell Health, Manorville, USA; 4 Neurosurgery, OhioHealth Grant Medical Center, Columbus, USA; 5 Neurosurgery, Massachusetts General Hospital, Boston, USA

**Keywords:** global neurosurgery, metallic arrowhead, neurosurgical management, neurotrauma, penetrating brain injury, sub-saharan africa

## Abstract

This case report and literature review describe an intracranial injury resulting from a handcrafted arrow that required emergent surgical management in a patient treated at a public hospital in Sub-Saharan Africa. The authors are members of the non-profit organizations Chunilal Initiative and Kisumu Intuitive, which deliver charitable neurosurgical care to the region on a quarterly basis. This unique neurotrauma case, involving a penetrating, contaminated, metallic object, highlights the surgical and antibacterial management of intracranial arrow injuries and emphasizes the importance of neurosurgical missions to underserved regions globally. The patient is a 16-year-old male tribal member with no known or reported comorbidities. He presented with a Glasgow Coma Scale of 15 and imaging consistent with intracranial penetration adjacent to the frontal sinus by a handcrafted Maasai Mara arrow. Due to the barbed nature of the arrow, the frontal bone was drilled to allow for the removal of the arrow. The dura was repaired, and the wound was irrigated without intraoperative or postoperative bleeding complications. The arrow was inoculated with animal feces. Prophylaxis treatment with gentamicin was warranted before the surgery, and an additional three doses were given every eight hours in the next 24 hours. On postoperative day one, periorbital edema was stable, and the neurological exam was grossly intact. The patient was discharged with an uneventful course and prescribed metronidazole for fourteen days. He followed up three weeks later, when the patient had a well-healed incision and an intact neurological exam; however, he did not attend his three-month follow-up. Overall, a teenage boy with an unknown medical history presented to the Jaramogi Oginga Odinga Teaching and Referral Hospital in Kenya, Africa, with a penetrating intracranial injury requiring emergency neurosurgical intervention. The successful management of intracranial arrow injuries in a resource-limited setting highlights the importance of surgical expertise, infection control, and interdisciplinary care in neurotrauma cases. This case highlights the importance of ongoing investment in neurosurgical training, infrastructure, and access to care in underserved regions, such as Sub-Saharan Africa.

## Introduction

Penetrating head injuries (PHIs), though rare, consist of only 0.4% of all head injuries, pose an exceedingly high risk to patients, and are potentially life-threatening [1]. PHIs are defined as injuries that breach the skull and protective barriers of the brain, such as gunshot wounds, stabbings, and accidents with sharp objects [2]. Intracranial complications after PHIs include brain abscess, meningitis, cerebrospinal fluid (CSF) leakage, hemorrhage, neurological deficits, and mortality [3]. Coating arrows in animal feces is a common practice in Sub-Saharan Africa, although it is poorly documented in the literature. This report describes a case of PHI induced by a contaminated metallic object in Sub-Saharan Africa, which required urgent neurosurgical intervention. It highlights the importance of documenting such cases, as the literature on the topic is sparse. The authors are part of the non-profit organizations Chunilal Initiative and Kisumu Intuitive, which provide charitable neurosurgical care to the region on a quarterly basis. These efforts are part of a broader global neurosurgery movement aimed at addressing disparities in access to specialized surgical care in low-resource settings, exemplifying how sustained international collaboration can improve outcomes in underserved communities.

## Case presentation

**Figure 1:**
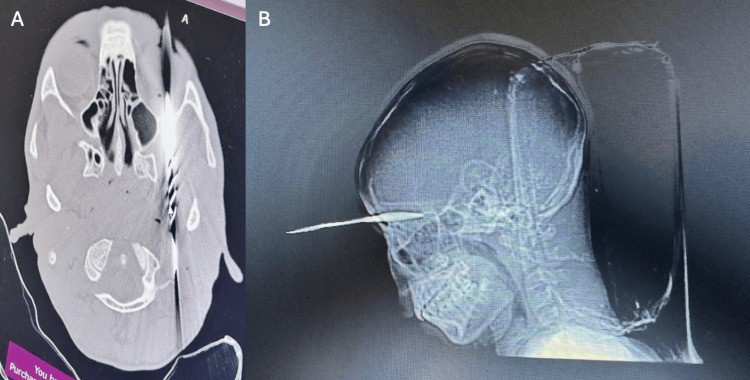
(A) Axial view and (B) sagittal view of the CT scan showing the arrowhead CT: computed tomography

**Figure 2:**
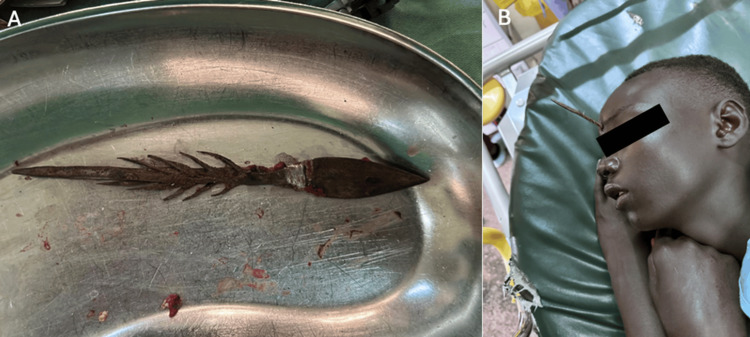
(A) The arrowhead removed from the patient's skull. (B) The arrowhead upon presentation, embedded into the skull. Red arrow indicates entry point of arrow

A 16-year-old boy presented with a frontal parasagittal PHI adjacent to the frontal sinus from a homemade arrow (Figures 1-2). The patient was struck by a metallic arrow from approximately 100 yards away. Upon presentation, the patient was neurologically intact with extreme periorbital edema and a Glasgow Coma Scale of 15. The patient presented to the Jaramogi Oginga Odinga Teaching and Referral Hospital in Kenya, Africa. A CT scan was performed. The patient had a hypotrophic frontal sinus, which was not disrupted. The patient received a tetanus shot upon admission. Due to the design of the arrow, heavily barbed, it was firmly embedded in the anterior fossa.

The patient was given IV gentamicin 60 mg 30 minutes before the surgery. After the patient was prepped and draped in a sterile fashion, an elliptical incision was made around the entry point with excisional debridement of the scalp and pericranium adjacent to the arrow. Due to the position of the arrow within the skull coordinated with imaging, a frontal craniotomy was carried out, keeping the arrow central to the bone window. The entry point was followed to the anterior fossa point of fixation. The arrow was removed with direct traction without incident or vascular injury. No intracranial bleeding was noted. The macerated dura was repaired with a small piece of pericranium harvested from the same incision.

The wound was irrigated with hydrogen peroxide and gentamicin-infused saline and then closed with an interrupted Vicryl suture. There were no immediate complications, no CSF leak, and no rhinorrhea; therefore, the decision was made to proceed no further. As the Maasai people coat their arrows in dung from cattle, the risk of infection is extremely high. The patient was therefore treated with IV gentamicin, 60 mg in three doses, every eight hours, over the next 24 hours. On postoperative day 1, periorbital edema was stable, and the neurological exam was grossly intact. However, due to the patient’s eyes being swollen shut, there was no ability to test cranial nerve II. The patient remained stable with no signs of infection. The surgical removal of the arrow was considered successful as the penetrating object was removed, the injury was surgically repaired, and the patient remained stable while in the hospital. The patient returned to his tribe with a prescription of metronidazole 500 mg taken two times a day every twelve hours for fourteen days. The patient returned at three weeks for a follow-up. The patient presented with no signs of infection, a well-healed incision, and an intact neurological exam, including cranial nerve II. The patient did not attend his three-month follow-up.

## Discussion

PHIs involving metallic objects, although rare, have previously been documented. Comprehensive imaging, including CT and possibly digital subtraction angiography, is essential to evaluate the extent of the foreign object’s trajectory and identify any vascular injuries. MRI should be avoided due to the risk of ferromagnetic effects on metallic fragments [4,5]. The primary treatment is surgical removal of the foreign object. This involves a craniotomy to access and safely extract the object while minimizing further brain damage. Techniques such as corticotomy through a shorter tract can be employed to reduce brain injury and manage vascular injuries under direct vision [4-6]. During surgery, meticulous hemostasis is crucial to control bleeding. Debridement of necrotic tissue and thorough irrigation are also essential steps to reduce the risk of infection [4-6]. Postoperative management includes prophylactic antibiotics to prevent infection and anticonvulsants to minimize the risk of seizures. Long-term follow-up with imaging is necessary to monitor for complications such as brain abscesses or delayed hemorrhage [4,5]. A team involving neurosurgeons, radiologists, and critical care specialists is essential for optimal outcomes. This approach ensures comprehensive care from initial assessment through postoperative management [7].

PHIs are associated with a significant risk of intracranial infections, including meningitis and brain abscesses. The Infectious Diseases Society of America highlights that the risk of infection is exceptionally high in cases involving depressed cranial fractures and CSF leaks [8]. In civilian practice, the persistence of intraparenchymal osseous or metallic fragments, projectile trajectory through contaminating cavities, and prolonged hospitalization were identified as independent risk factors for intracranial infections [9]. In this patient, the risk of infection was extremely high due to the Maasai people coating their arrows in bovine feces. As such, he should have been started on the prophylactic antibiotic regimen deemed most appropriate, but records to this effect are unclear. Antibiotic prophylaxis administered to patients has been shown to significantly reduce infection rates compared to patients who do not receive prophylactic treatment [10]. Additionally, follow-up was challenging, as the patient lived a considerable distance from the hospital and was unable to attend subsequent visits.

There is a big concern for postsurgical complications beyond infections. A meta-analysis from January 2010 to November 2022 revealed that out of 24,136 patients across 19 studies of postoperative complications in Sub-Saharan Africa, 24,136 patients experienced a postoperative complication. It also highlighted that these complications are exaggerated by structure-related factors, such as access to follow-ups and adherence to protocols [11].

There are significant disparities in neurosurgical care across Sub-Saharan Africa, highlighting critical challenges in access, infrastructure, and workforce capacity, regardless of ongoing efforts to address these gaps. Despite these challenges, considerable progress has been made, primarily driven by international collaborations, regional training programs, and the dedication of local neurosurgeons to provide patient care that is desperately needed. Collaborative international programs have been essential in enhancing neurosurgical education and training. Notable initiatives include the Neurosurgery Education and Development Foundation, the Weill Cornell Tanzania Neurosurgery Project, and the Duke East Africa Neurosurgery Program. These programs have successfully increased the number of trained neurosurgeons in the region [12].

Although there is improvement, the neurosurgeon-to-population ratio remains low. For instance, Sub-Saharan Africa has approximately one neurosurgeon per 2.62 million inhabitants, with significant disparities between countries. As of 2023, there are 1165 neurosurgeons reported on the continent, but there is an unequal distribution of where they are located; only 409 out of the 1165 neurosurgeons located in Africa reside in Central, East, and West Africa [13]. This leaves a significant deficit, as the population in these areas is roughly 1.16 billion [14]. The disparity between neurosurgeons and citizens is quite jarring, highlighting the urgent need for international neurosurgical partnerships and collaborations. Yet there is a need for increased institutionalization training in Africa.

Efforts to train more neurosurgeons locally, such as through the World Federation of Neurosurgical Societies Rabat Training Center, have been beneficial, with graduates returning to practice in their home countries [15]. Another strategy to increase the availability of neurosurgical services in underserved regions within Sub-Saharan African countries is to establish new residency training programs outside of cosmopolitan cities, where existing programs may already exist [16]. The caseload of these rural hospitals would allow for ample training of competent surgeons, enabling them to stay in their country of origin.

Access to essential neurosurgical equipment also remains a challenge. While CT scanners are available to 86% of surveyed neurosurgeons, MRI availability is limited to 38%, and advanced tools like neuronavigation are virtually absent in Sub-Saharan African countries [15]. This lack of equipment dampens the ability to provide comprehensive neurosurgical care. The field faces numerous challenges, including limited resources, inadequate infrastructure, and financial barriers that hinder patient access. However, ongoing efforts are being made to address these issues through the expansion of training programs, international collaborations, and the adoption of affordable, low-maintenance technologies [15,17].

To further improve neurosurgical care, enhancing training capacity, particularly in underserved regions, and developing sub-specialization programs is crucial. Additionally, establishing electronic medical databases and fostering international research collaborations are essential for advancing the field [18]. While significant strides have been made in neurosurgery in Sub-Saharan Africa, continued efforts are necessary to address the persistent challenges and establish a sustainable and self-sufficient neurosurgical infrastructure.

Penetrating arrow injuries, similar to the one in this case report, have been documented, although with variations in the penetration [19-21]. Each case reported successful removal of the arrow and positive acute care outcomes; however, there remained little to no documentation of long-term follow-up. The use of community health workers (CHWs), mobile health (mHealth) technologies, and scheduled outreach clinics offers a possible sustainable solution to this issue. CHWs can be trained in postoperative care and possess the necessary tools and knowledge to closely monitor patients. The use of Yendanafe mHealth in rural Malawi included patient-centered care, scheduling, reminders, and follow-up appointments with patients [22]. Likewise, the M-JALI (Mobile-Jamii Afya Link) mobile application, used in Kenya, provided 3,900 CHWs with the ability to register homes, make healthcare referrals, and record and report data on their patients [23]. In Zambia, CHWs were able to have household health visits, write referrals, and follow up with patients [24]. Enacting a program like this for the Maasai Mara and other tribes may be crucial to the sustainability of neurosurgical practices in the region. Even though many of these tribes lack the technological resources, a CHW traveling to the patient to either video chat with the provider or record an update on these patients may be a very realistic move forward.

The case presented here specifically highlights the successful management of a rare and high-risk PHI caused by a metallic arrow in a resource-limited setting in Sub-Saharan Africa. Despite the significant challenges posed by the remote location, limited medical infrastructure, and heightened risk of infection due to the cultural practice of coating arrows in bovine dung, the patient underwent a meticulous and effective neurosurgical procedure with a positive short-term outcome. This report highlights the importance of a comprehensive understanding of neuroanatomy, surgical expertise, and effective antimicrobial treatment in managing such injuries. Furthermore, it emphasizes the critical need for expanded neurosurgical training, improved access to medical resources, and the development of systems for follow-up care in underserved regions. While international collaborations and training initiatives have made notable progress in addressing disparities in neurosurgical care across Sub-Saharan Africa, this case serves as a reminder of the ongoing challenges in providing equitable and sustainable healthcare. The ability to perform complex neurosurgical interventions under constrained conditions is a testament to the dedication of local healthcare providers and the necessity for continued investment in regional neurosurgical infrastructure and education.

## Conclusions

The treatment of this patient was considered successful, as the arrow was removed and the patient remained stable, returning to his tribe. The patient returned for a three-week follow-up, showing no signs of infection, a well-healed incision, and an intact neurological examination, including cranial nerve II. This case underscores the capability to perform high-risk neurosurgical procedures under resource-limited conditions, highlighting the importance of a comprehensive understanding of anatomy, physiology, and infection management in ensuring patient survival. However, the absence of long-term follow-up care illustrates the challenges of limited healthcare access in remote regions. It emphasizes the need for sustainable healthcare infrastructure and systems to provide continuity of care for patients in underserved areas such as Sub-Saharan Africa.
